# A commentary on ‘Comparison of surgical outcomes and learning curve for robotic versus laparoscopic living donor hepatectomy: a retrospective cohort study’ (Int J Surg 2022;108:107000)

**DOI:** 10.1097/JS9.0000000000000350

**Published:** 2023-06-05

**Authors:** Dingwei Xu, Jie Huang

**Affiliations:** Department of Hepatopancreatobiliary Surgery, The Second Affiliated Hospital of Kunming Medical University, Kunming, Yunnan, People’s Republic of China

*Dear Editor*,

Living donor liver transplantation (LDLT) has emerged as an alternative method for some patients with end-stage liver disease to overcome the limitations of deceased donor organ shortages^1^. In 2002, Cherqui *et al*.^2^ reported the first case of pure laparoscopic living donor hepatectomy (resection of the left lateral lobe) for LDLT in children, demonstrating its safety and feasibility. Additionally, Chen *et al*.^3^ reported the first small series of 13 robotic living donor right hemihepatectomy (RLDRH) cases in 2016, demonstrating the safety and feasibility of the robotic approach. However, due to the technical complexity, a totally minimally invasive approach for living donor right hepatectomy remains a significant challenge for transplant surgeons.

Compared to the conventional laparoscopic approach, robotic surgery overcomes limitations in range of motion and physiological tremors, such as an unstable camera, rigid instruments and a loss of 3D vision. We congratulate the authors on their comprehensive work comparing the surgical outcomes of robotic versus laparoscopic living donor hepatectomy at a single institution^4^. Despite recent advancements in robotic technology, large-volume experience in LDLT with a minimally invasive approach is still limited mainly to Asia and the Middle East, and there are few surgeons who can perform both laparoscopic living donor left hemihepatectomy (LLDRH) and RLDRH. The current study adds to the existing evidence on RLDRH surgical outcomes and learning curves. However, we have several concerns about this study, which are detailed below.

First, a learning curve refers to the process of acquiring proficiency in a task or skill over time through practice and experience. In surgery, the learning curve represents the amount of time it takes for a surgeon to become proficient in a particular procedure. As surgeons perform more surgeries, their proficiency improves, resulting in better surgical outcomes. The shape of the learning curve can vary, with some procedures having a steeper learning curve than others, depending on factors such as procedure complexity and the surgeon’s experience. The learning curve can also be influenced by other factors, such as the surgeon’s training and the availability of resources and support. Based on the author’s research, we can assume that the author’s institution is a large-volume LDLT centre. Until March 2022, a total of 1040 living donor liver transplants were performed. In such a large number of surgical operations, whether the personal learning curve is universal is still debatable.

Second, the author seems to ignore some details, such as whether the surgical team was stable. In surgery, the relationship between the individual learning curve and the team learning curve is interrelated. An individual’s learning curve represents a personal journey to mastery of a surgical procedure, while a team’s learning curve reflects the group’s ability to collaborate to achieve optimal outcomes. In some cases, a team’s learning curve may be steeper than an individual’s due to the sharing of knowledge, skills and experiences. Conversely, if an individual’s learning curve is steep, it may contribute to a faster team learning curve as the individual can share their knowledge with the team.

Third, the relationship between the learning curve in surgery and the interval between surgeries is complex. We believe that the frequency of surgery intervals to reach the learning curve is more important than the number of cases. As surgeons perform more surgeries, their proficiency and surgical outcomes improve, resulting in a shorter learning curve. However, the interval between surgeries influences this process. If the interval between surgeries is too long, the surgeon may need to spend more time recalling the procedure, resulting in a longer learning curve. Conversely, if the interval between surgeries is too short, the surgeon may become fatigued, resulting in poor surgical outcomes.

In conclusion, we would like to thank the authors for their first report comparing surgical outcomes of LLDRH and RLDRH in a single centre. In teams with high expertise and with appropriately selected living donors, robotic donor hepatectomy can be performed safely and effectively. However, further research is needed to adequately evaluate the impact of a robotic hepatectomy.

## Conflicts of interest disclosure

No potential financial or non-financial conflicts of interest.
